# Angiostatic treatment prior to chemo- or photodynamic therapy improves anti-tumor efficacy

**DOI:** 10.1038/srep08990

**Published:** 2015-03-11

**Authors:** Andrea Weiss, Débora Bonvin, Robert H. Berndsen, Edoardo Scherrer, Tse J. Wong, Paul J. Dyson, Arjan W. Griffioen, Patrycja Nowak-Sliwinska

**Affiliations:** 1 Institute of Chemical Sciences and Engineering, Swiss Federal Institute of Technology (EPFL), Lausanne, Switzerland; 2 Angiogenesis Laboratory, Department of Medical Oncology, VU University Medical Center Amsterdam, The Netherlands

## Abstract

Tumor vasculature is known to be poorly organized leading to increased leakage of molecules to the extravascular space. This process can potentially increase interstitial fluid pressure impairing intra-tumoral blood flow and oxygen supply, and can affect drug uptake. Anti-angiogenic therapies are believed to reduce vascular permeability, potentially reducing interstitial fluid pressure and improving the extravasation of small molecule-based chemotherapeutics. Here we show that pretreatment of human ovarian carcinoma tumors with sub-optimal doses of the VEGFR targeting tyrosine kinase inhibitor axitinib, but not the EGFR targeting kinase inhibitor erlotinib, induces a transient period of increased tumor oxygenation. Doxorubicin administered within this window was found to enter the extravascular tumor space more rapidly compared to doxorubicin when applied alone or outside this time window. Treatment with the chemotherapeutics, doxorubicin and RAPTA-C, as well as applying photodynamic therapy during this period of elevated oxygenation led to enhanced tumor growth inhibition. Improvement of therapy was not observed when applied outside the window of increased oxygenation. Taken together, these findings further confirm the hypothesis of angiostasis-induced vascular normalization and also help to understand the interactions between anti-angiogenesis and other anti-cancer strategies.

After reaching a critical size, the growth of a tumor becomes dependent on its ability to secure a blood supply through the development of a vascular network^1^. This process is mediated by the release of angiogenic growth factors by tumor cells in response to a hypoxic tumor microenvironment, initiating the growth of new blood vessels, i.e. angiogenesis^1^,^2^,^3^. The presence of excessive pro-angiogenic growth factors, however, results in a phenotypically aberrant tumor vasculature characterized by dilatation, tortuosity and increased permeability. Such leakiness leads to protein extravasation and blood clotting, which impairs blood flow and induces high interstitial fluid pressure (IFP) in the tumor^4^,^5^. Combined, these features contribute to poor perfusion in tumors and consequently decreased uptake of chemotherapeutics^6^. In addition, they can result in reduced treatment benefits of oxygen-dependent therapies such as radiotherapy^7^ and photodynamic therapy^8^. Moreover, many anticancer drugs are less effective in hypoxic environments, which can exacerbate drug resistance^9^.

Several approaches have been developed to enhance intra-tumoral drug delivery, e.g. low-dose PDT^10^, the application of a type I collagenase inhibitor^11^ and the use of various anti-angiogenic agents^12^,^13^,^14^. These treatments result in a transient period where the morphology of the tumor vasculature appears more normal enabling improved drug delivery. Moreover, it is well documented that appropriately tailored combined therapies show synergism or additive anti-cancer effects^15^,^16^,^17^,^18^. Anti-angiogenic drug therapy is of particular interest as it can counteract the upregulation of pro-angiogenic factors characteristic of the tumor microenvironment and, as a form of anticancer therapy, ultimately aims to starve tumor growth through the inhibition of blood vessel growth^3^. It has been shown that anti-VEGF, anti-VEGFR, or non-VEGF(R) targeting angiostatic compounds can temporarily reduce the diameter of enlarged vessels and vessel tortuosity in time and dose dependent manners, and can normalize the basement membrane^19^,^20^. Moreover, angiostatic therapy has been shown to reduce vessel pore size leading to reduced interstitial fluid pressure and restore convective drug penetration, facilitating the delivery and uptake of small molecules (below 12 nm in diameter), while simultaneously hindering the delivery of larger species^6^,^12^,^21^. As a consequence, intra-tumoral blood flow may increase giving rise to enhanced tumor oxygenation. This anti-angiogenesis induced transient window of tumor blood vasculature normalization has been exploited to apply other chemotherapeutics, at the appropriate time and dose, leading to improved treatment outcomes^22^,^23^. It should be noted, however, that there is controversy over the existence of an angiostasis-induced normalization window, and some groups have reported on absence of increased drug delivery following angiostatic drug treatment^24^.

In the present study, we determined the window during which a transient increase of intra-tumoral oxygenation, referred to as the normalization window, takes place following treatment with targeted angiostatic tyrosine kinase inhibitors (TKIs) in A2780 human ovarian carcinoma xenografts implanted on the chicken chorioallantoic membrane (CAM) model. Transient oxygenation enhancements in the tumor were obtained after intravenous administration of the VEGFR targeting small molecule TKIs axitinib (Inlyta®) and sunitinib (Sutent®), but not following treatment with the EGFR targeting inhibitor erlotinib (Tarceva®). We subsequently used the axitinib-induced normalization window for combination therapy with PDT, as well as with two small molecule-based chemotherapeutics, i.e. doxorubicin (8 nm in diameter) and [Ru(η^6^-*p*-cymene)Cl_2_(pta)], where pta = 1,3,5-triaza-7-phosphaadamantane (RAPTA-C; 1 nm in diameter), a ruthenium-based anti-cancer and anti-metastatic drug under development^25^,^26^,^27^. In all the combination therapies evaluated, improved anti-tumor efficacy was observed when the treatment was performed during the axitinib-induced normalization window, compared to tumors treated with the same compounds without an axitinib pretreatment or when applied outside the normalization window.

## Results

### Axitinib and sunitinib at suboptimal doses induce transiently increased tumor oxygenation

**Figure 1:**
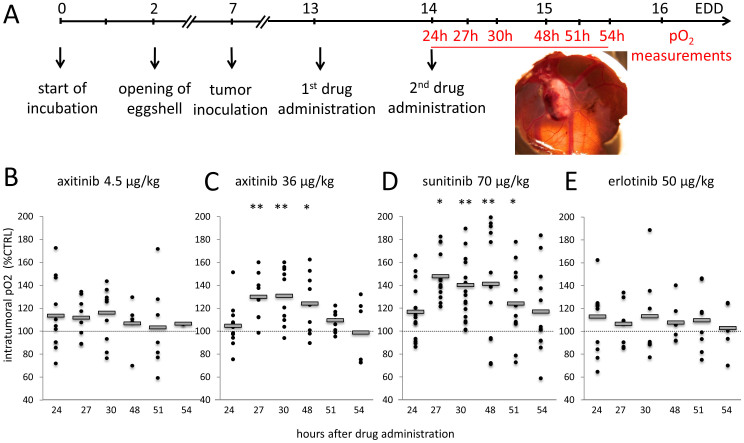
Oxygen partial pressure (pO_2_) measured in tumors treated with axitinib, sunitinib and erlotinib. (A). Time schedule of pO_2_ measurements (shown in red) after two intravenous injections of (B) axitinib (4.5 μg/kg, n = 2–11), (C) axitinib (36 μg/kg, n = 6–12; **p = 0.004 and 0.003, *p = 0.03 for 27 h, 30 h and 48 h, respectively), (D) sunitinib (70 μg/kg, n = 9–15; *p < 0.04 (27 h), **p = 1.7E − 7 (30 h), **p = 3.5E − 4 (48 h), and *p = 0.015 (51 h), or (E) erlotinib (n = 5–10). Values are normalized to the pO_2_ values measured in untreated tumors (% control).

Oxygenation of human A2780 ovarian carcinoma tumors, that are negative for EGFR, xenografted onto the chorioallantoic membrane of chicken embryos was studied following treatment with the anti-angiogenic tyrosine kinase inhibitors (TKIs) axitinib, sunitinib or erlotinib (Fig. 1A). Each drug was given i.v. at time t = 0 and followed by a second i.v. injection at 24 hours. Oxygen partial pressure (pO_2_) was measured in tumors at various time points after the first i.v. drug administration by inserting the pO_2_ probe into the tumor core. The TKIs were administered at the following doses: axitinib at 4.5 μg/kg (Fig. 1B) and 36 μg/kg (Fig. 1C), sunitinib at 70 μg/kg (Fig. 1D), and erlotinib at 50 μg/kg (Fig. 1E). The pO_2_ measurements in control tumors were performed at the same time points. Overall tumor growth was monitored over 7 days after the first TKI injection (Supplementary Fig. S1). At the applied drug doses, inhibition of A2780 tumor growth was sub-optimal, i.e. by approximately 7% (axitinib 4.5 μg/kg; p = 0.70; n = 26) and 40% (axitinib 36 μg/kg; p = 0.07; n = 15), 25% (sunitinib; p = 0.04; n = 7), and by approximately 28% (data for erlotinib 40 and 80 μg/kg; p = 0.32 and 0.02; n = 12 and 13, respectively).

Axitinib administered at low dose of 4.5 μg/kg (Fig. 1B) did not induce a significant increase in intratumoral pO_2_, measured between 24 and 54 hours after the first injection of axitinib. At a higher dose of axitinib (36 μg/kg), a significant increase in tumor oxygenation of 31% was observed, with a maximum identified at 30 hours after the first drug injection (Fig. 1C; **p < 0.01 at t = 27 h and t = 30 h; *p < 0.05 at t = 48 h). Sunitinib also induced a significant increase in tumor oxygenation between 24 and 48 hours after administration of the first drug leading to a maximal oxygenation increase of 47% at t = 27 h (Fig. 1C; *p < 0.05). Axitinib showed a more stable normalization window compared to sunitinib, with clear start and end points. Consequently, axitinib was used for the subsequent combination studies.

### The axitinib-induced vascular normalization window increases doxorubicin extravasation

**Figure 2:**
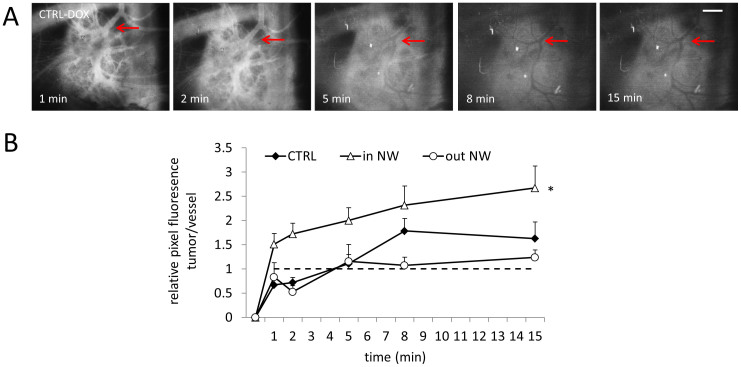
Effect of vascular normalization using axitinib on the uptake of DOX into the tumor tissue. (A). Representative fluorescence images of tumors after the administration of DOX (15 mg/kg). Red arrows highlight a representative tumor vessel where the extravasation of DOX is visible. (B). Quantification of DOX distribution as a function of time without an axitinib pre-treatment (‘CTRL-DOX'), with DOX administered in the axitinib-induced normalization window at t = 30 h (‘in NW') and with DOX administered before the axitinib-induced normalization window t = 24 h (‘out NW'). Images were taken at 4x magnification. White bar in the upper right corner represents 0.5 cm and is valid for all images. *p < 0.042 between ‘in NW' and ‘out NW'; n = 3–10.

To investigate whether the identified normalization window could be exploited to increase drug delivery, doxorubicin (abbreviated to ‘DOX') at 15 mg/kg was administered i.v. in a 100 μl bolus, within the normalization window (i.e. 30 hours post initial axitinib administration; referred to as ‘in NW') and before the normalization window (at 24 hours post initial axitinib administration; referred to as ‘out NW'). A control group received only DOX and was included to establish the distribution of DOX in non-pre-treated tumors. The ratio of DOX present in the tumor tissue versus the CAM vasculature was quantified using the intrinsic fluorescence of DOX (excited at λ_ex_ = 470 ± 20 nm; λ_em_ ≥ 520 nm), as previously reported^28^. Fluorescence images were acquired for each treatment group 1, 2, 5, 8 and 15 minutes post DOX injection (images of the ‘CTRL-DOX' group are provided in Fig. 2A, and red arrows indicate tumor blood vessels where the gradual extravasation of DOX becomes visible with time). An accelerated leakage of DOX from the tumor vasculature into the tumor parenchyma was observed when DOX was administered within the axitinib-induced normalization window (at 30 hours after the initial administration of axitinib). In contrast to the ‘CTRL-DOX' and ‘out NW' groups, extravasation of DOX was observed even after 1 minute in the ‘in NW' group. Despite the fact that DOX, once taken up into the tumor cells binds to DNA and loses its fluorescence, quantification of the fluorescence images indicates an increase in the rate of DOX leakage in the ‘in NW' group relative to the other groups. At the final time point of 15 minutes there is ca. 2.5-fold higher fluorescence in the tumor tissue than in the blood vessels in the ‘in NW' group, compared to an increase of only 1.5-fold in the ‘CTRL-DOX' group, see Figure 2B. Interestingly, the ‘out NW' group shows a lower ratio of fluorescence between the tumor and blood vessels, indicating that this treatment possibly reduces the leakage of DOX from the vessels into the tumor tissue, with an almost equivalent fluorescence at 15 min. The enhanced ratio in the ‘in NW' group is an underestimation of enhanced extravasation because of the above-mentioned DNA binding. Administration of DOX ‘out NW' did not increase the extravasation (p = 0.53 vs. CTRL).

### Tumor growth inhibition of doxorubicin and RAPTA-C in the axitinib-induced vascular normalization window

**Figure 3:**
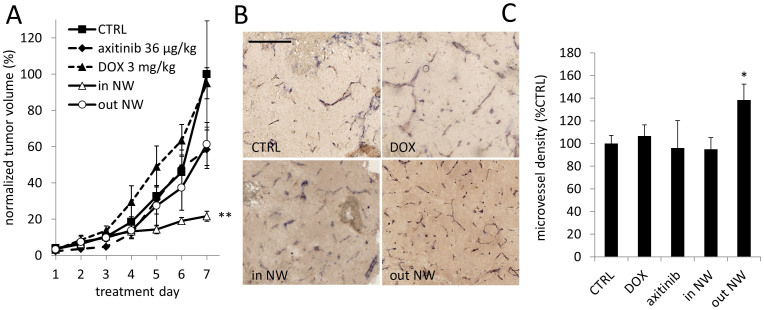
Enhanced effect of DOX after pretreatment with axitinib. (A). Tumor growth curves of the different treatment groups (n = 6–13). Axitinib (36 μg/kg) was administered on days 1 and 2 and DOX (3 mg/kg) was administered on day 2 at 24 h for ‘out NW', 27 h for DOX and 30 h for ‘in NW' (n = 6–24). Tumor size was monitored daily for 7 consecutive days (axitinib 36 μg/kg: n = 15, p = 0.07; ‘DOX 3 mg/kg': n = 10, p = 0.30; ‘in NW' vs. CTRL n = 9, **p = 0.005; ‘out NW' vs. CTRL: n = 7, p = 0.28, 2-way ANOVA). (B). Representative images of tumors resected on the last experimental day from each group stained for CD31. Images were taken at 100x magnification, the bar in the top left image representing 20 μm. (C). Quantification of the microvessels (n = 3–24). Error bars in (A) and (C) represent standard error of mean, significance indicated vs CTRL, and *p < 0.05 and **p < .01.

To test whether the increased drug delivery results in enhanced anti-tumor efficacy tumor growth experiments were performed. Tumors were transplanted onto the CAM and treatment was initiated on embryo development day (EDD) 11 in order to monitor tumor growth over a period of 7 days. The doxorubicin dose was reduced to 3 mg/kg, which is suboptimal to avoid lethality to the embryos. Tumor growth curves confirmed that anti-tumor efficacy of doxorubicin is strongly enhanced when administered in the normalization window (‘in NW') (Fig. 3A). Doxorubicin alone did not inhibit tumor growth and axitinib alone reduced growth by approximately 40% (p < 0.07 as compared to control). The combination therapy of giving DOX in the normalization window significantly improved efficacy and inhibited tumor growth by 78% (**p < 0.0001 vs. DOX; **p = 0.005 vs ‘out NW'). Interestingly, application of DOX outside the normalization window (‘out NW') did not improve the efficacy of the combination (Fig. 3A). These results are in accordance with the extravasation of DOX to the tumor tissue (Fig. 2B). Immunohistochemical staining with the CD31 antibody showed that the suboptimal doses of drugs did not significantly change the microvessel density (MVD) in this experiment.

**Figure 4:**
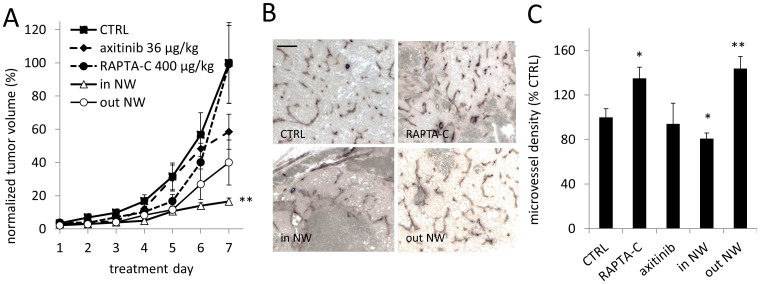
Enhanced effect of RAPTA-C on tumor growth after prior treatment with axitinib. (A). Tumor growth curves. Administration of axitinib on day 1 and 2 and RAPTA-C on day 2 (n = 6; **p = 0.0015 for ‘in NW' vs. CTRL; p = 0.08 for ‘out NW' vs. CTRL, and p = 0.07 for axitinib, 2-way ANOVA). (B). Representative CD31 staining images. Images were taken at 200x magnification; the bar in the top left image represents 10 μm. (C). Quantification of microvessel density (represented as a percentage of the control, n = 4–18). Error bars in (A) and (C) represent standard error of the mean, significance is indicated vs. CTRL, and *p < 0.05 and **p < 0.01.

Enhanced tumor growth inhibition was also demonstrated for the experimental ruthenium-based compound RAPTA-C. RAPTA-C treatment at 400 μg/kg did not affect tumor growth when administered alone (Fig. 4A; n = 7, p = 0.38). When applied in combination with axitinib (‘in NW'), this ineffective dose of RAPTA-C significantly inhibited tumor growth by 83.5% compared to the control group (n = 6; **p = 0.0015; **p = 0.0005 vs. ‘out NW'). This treatment was also more effective than the ‘out NW' treatment, which inhibited tumor growth by approximately 60% (n = 7, p = 0.08 vs. CTRL). Microvessel density assessment showed that the ‘in NW' group had a slight but significantly reduced MVD as compared to control tumor (*p < 0.048, n = 15–18). RAPTA-C itself showed a slight but significant increase in MVD, which was not changed by the application of axitinib outside the normalization window (*p = 0.011 and **p = 0.003, n = 11 and n = 19 for ‘RAPTA-C' and ‘out NW' groups, respectively).

In order to understand the improved anti-tumor efficacy seen when combining axitinib with DOX or RAPTA-C *in vivo*, these compounds were tested separately or in combination *in vitro* on immortalized human endothelial (ECRF24) cells and human ovarian carcinoma (A2780) cells. As shown in Supplementary Table S1, two doses of each compound were identified to inhibit cell proliferation by 40% or less, mimicking *in vivo* conditions. The combinatory index (CI) was calculated for each pair of compounds in each cell line based on *in vitro* single drug and combination efficacies and was used as an indicator of synergy (CI < 0.8). These results showed that, under the conditions tested, axitinib in combination with doxorubicin did not result in a synergistic inhibition of cell proliferation in either of the cell lines (CI > 0.8), while the combination of axitinib with RAPTA-C led to a synergistic interaction in the ECRF24 cells but only an additive (CI = 1) or even slight antagonistic (CI = 1.3) effect in A2780 cells.

### Effect of low-dose PDT on tumor growth following pretreatment with axitinib

**Figure 5:**
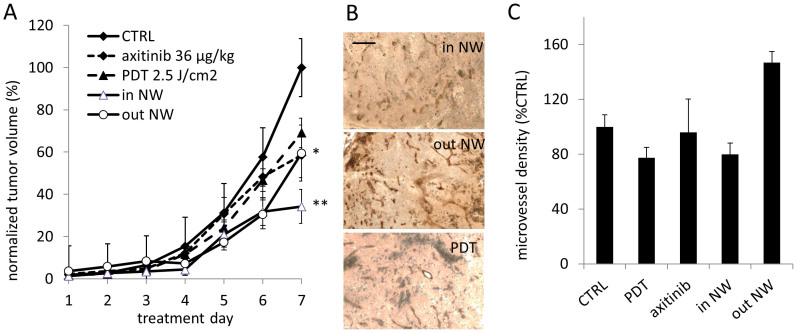
Effect of low-dose PDT on tumor growth following vascular normalization with axitinib. Normalized A2780 tumor volume (% control) monitored between treatment day 1 to 7. (A). Tumors were treated with Visudyne®-PDT (2.5 J/cm^2^; n = 36), axitinib (36 μg/kg; n = 15), or the combination of both performed within (‘in NW' vs. CTRL; **p = 0.0001, n = 15) or before (‘out NW' vs. CTRL; *p = 0.04, n = 8, 2-way ANOVA) tumor oxygenation enhancement. (B). Representative images of tumor from each group stained for CD31 in tumors resected on the last experimental day. Images were taken at 200x magnification, the bar in the top right image represents 10 μm. (C). Quantification of the MVD represented as a percentage of the control (n = 3–18). Error bars in (A) and (C) correspond to the standard error of mean, significance indicated vs CTRL, and *p < 0.05 and **p < 0.01.

To investigate whether the axitinib-induced normalization window could potentiate tumor oxygenation dependent therapies, the effect of PDT was evaluated (Fig. 5). In order to define PDT conditions leading to suboptimal tumor growth inhibition, A2780 tumors were first treated with Visudyne®-PDT (0.2 mg/kg embryo weight; λ_ex_ = 420 ± 20 nm, drug-light interval 1 min) at various low light fluencies (1, 2.5 or 5 J/cm^2^, irradiance 28.5 ± 1.7 mW/cm^2^). At 2.5 J/cm^2^ tumor growth was inhibited non-significantly by 30% (Supplementary Fig. S2).

While Visudyne®-PDT and axitinib (36 μg/kg) inhibited tumor growth by 30% (*p = 0.014) and 40% (p < 0.07), respectively, a reduction of tumor growth by 66% (n = 15, **p < 0.0001, compared to the axitinib group) was observed when treating tumors with Visudyne®-PDT at 30 hours after the first injection of axitinib (‘in NW'; Fig. 5A). Application of PDT out of the normalization window (‘out NW') did not show enhanced effects compared to axitinib alone. Immunohistochemical staining with the CD31 antibody (Figure 5B) showed that the Visudyne®-PDT applied ‘in NW' resulted in decrease of the microvessel density (MVD), see Fig. 5C.

Visudyne®-PDT was also combined with a lower axitinib dose (4.5 μg/kg, where no significant increase in tumor oxygenation was measured (Fig. 1B) applied at the same treatment schedules (Supplementary Fig S2B). Performing PDT at 30 hours after the first axitinib administration (‘in NW') slightly enhanced the treatment efficacy (41% tumor growth inhibition; p = 0.11 vs. CTRL, n = 7) compared to the ‘out NW' group (23% tumor growth inhibition; p = 0.788 vs. ‘in NW', n = 15). The overall axitinib dose in the ‘in NW' group was then increased to 13.5 μg/kg by administering axitinib for the third time 24 hours after PDT. This improved the treatment efficacy by 23% compared to the ‘in NW' group (Supplementary Fig S3; ‘in NW 3 injections'; 64% tumor growth inhibition; n = 8). The tumor growth inhibition of the ‘in NW 3 injections' group was comparable with the anti-tumor effect of PDT applied in the normalization window induced by axitinib at 36 μg/kg (total dose of 72 μg/kg). Interestingly, shifting the first axitinib injection in the ‘out NW' group to 4 hours after PDT (Supplementary Fig. S3; ‘out NW (4 h)'; 25% tumor growth inhibition; p = 0.62, n = 10) resulted in similar tumor growth inhibition in comparison to injecting the drug right after PDT (‘out NW'). These treatments did not provide better overall anti-cancer effect than the one observed with using normalization window. These results along with the intra-tumoral oxygenation measurements (Fig. 1) suggest that the timing between the axitinib administration and the PDT treatment, as well as the dose of the anti-angiogenic drug administered are both crucial for optimally exploiting the normalization window.

## Discussion

The effect of a pretreatment step was studied with the tyrosine kinase inhibitor (TKI), axitinib, on intra-tumoral delivery of small molecule-based chemotherapeutics on human ovarian carcinoma (A2780) xenografted on the chicken chorioallantoic membrane (CAM) model. Exposure to axitinib and sunitinib results in a period of enhanced oxygenation of the tumor. Axitinib induces a normalization window that leads to enhanced drug delivery as well as to improved efficacy of these drugs. Moreover, axitinib pretreatment potentiates the efficacy of vaso-occlusive Visudyne®-photodynamic therapy, a treatment modality that depends on sufficient oxygenation of the tumor tissue.

A variety of pre-clinical and clinical evidence is available that both supports and refutes the existence of a normalization window induced by angiostatic therapy. Various groups have reported measurable increases in tumor oxygenation, improved drug delivery, and effects on vessel function and morphology (permeability and/or blood flow), as indicators of vascular normalization after treatment with various angiostatic agents^29^. Increased oxygenation has been reported following the administration of bevacizumab^13^,^30^, tyrosine kinase inhibitors, e.g. sunitinib^31^, semaxanib^32^, a PI3K inhibitor^33^, EGFR inhibitors^33^,^34^, the cox-2 inhibitor apricoxib^35^, as well as following other angiostatic therapies, such as metronomic chemotherapy^36^. Enhanced delivery of doxorubicin has also been shown following the overexpression of platelet-derived growth factor-D (PDGF-D) in breast carcinoma^37^. Clinical evidence that supports vascular normalization includes reduced permeability and reduced tumor-associated edema^38^,^39^ following treatment with the pan-VEGFR inhibiting TKI cediranib in patients with recurrent gliblastoma, as well as reduced interstitial fluid pressure and improved delivery of fluorodeoxyglucose following bevacizumab treatment in rectal carcinoma^40^.

On the other hand, there are studies suggesting that the improved efficacy of angiogenesis inhibitors with other chemotherapeutics, does not involve enhanced oxygenation or drug delivery, and some even indicate that combinations are sometimes not beneficial^24^,^41^,^42^,^43^. In a clinical setting, Van der Veldt et al. reported reduced perfusion and a reduced net influx rate of docetaxel in patients with non-small cell lung cancer (NSCLC) following bevacizumab therapy (15 mg/kg), noticeable at 5 hours and lasting for 4 days^24^. In a preclinical study where axitinib was administered (10, 25 or 50 mg/kg once daily by oral gavage) 1 hour before or 1 hour after radiotherapy in mice implanted with human prostate tumors, it was shown that after the combination therapy the tumor vasculature was not functionally normalized and an increase in intra-tumor hypoxia was observed^41^,^42^. In another study axitinib (25 mg/kg injected intraperitoneally once daily) induced tumor hypoxia and seemed to suppress tumor blood perfusion, thereby decreasing the uptake of the tested chemotherapeutic drug in mice xenografted with rat gliosarcoma cells^43^. However, in these studies an axitinib induced normalization window was not clearly identified, and direct measurements of intra-tumor oxygen levels after axitinib treatment were not performed. More recently, the effect of low-dose axitinib (2–25 mg/kg administrated orally twice daily) was evaluated in combination with cyclophosphamide and a significantly increase in tumor retention of the chemotherapeutic drug was observed, indicating that the dosage and timing of drug administration is critical in identifying a normalization effect^44^.

While it is clear that there is still debate about the existence, the nature and the benefits of angiostasis-induced vascular normalization, in our studies the measured intratumoral oxygen partial pressure (pO_2_) was used as an indicator of tumor normalization. It was assumed that an increase in the pO_2_ is linked to a decrease in the interstitial fluid pressure and temporal vascular modulation, i.e. *normalization*. As previously described, such a normalization reduces the pore size on vasculature in solid tumors limiting vessel permeability for molecules larger than 12 nm in diameter, but enabling smaller-sized molecules to enter tumor space more easily/rapidly^12^. We subsequently found that administration of small molecule chemotherapeutics, i.e. doxorubicin (8 nm in diameter) or RAPTA-C (1 nm), during this normalization period led to a significant enhancement of extravasation of these drugs and improved overall tumor growth inhibition. Since RAPTA-C does not have an intrinsic fluorescence it could not be detected *in vivo* and therefore was administered at the same time schedule as DOX. Interestingly, RAPTA-C administered at lower dose (0.4 mg/kg) than DOX (3 mg/kg) was a more potent inhibitor of tumor growth. This finding is quite remarkable as RAPTA-C has low toxicity and when used as a monotherapy requires doses of 100 mg/kg injected daily over a number of days to have a similar effect on tumor inhibition^26^. Based on histology and *in vitro* experiments in ECRF24 endothelial cells it would appear that RAPTA-C inhibits tumor growth via both vascular modulation/normalization and anti-angiogenic^25^ mechanisms.

In our experimental regimen we were unable to induce a normalization window following treatment with erlotinib, the EGFR inhibitor, at 50 μg/kg, a dose that inhibits tumor growth by approximately 28%. This effect is almost equivalent to the efficacy of the dose used for sunitinib (inhibiting tumor growth by 25% vs. CTRL). It is conceivable that an increase in tumor oxygenation could appear beyond the time frame used in the study. The identification of a normalization window following erlotinib administration in mice bearing xenografts of head and neck squamous cell carcinoma (SQ20B) cells has been reported^34^. A possible explanation for these differing observations may be due to the sensitivity of tumor types to EGFR inhibition, as head and neck tumors frequently overexpress EGFR^45^ and the A2780 ovarian carcinoma cell line used for xenografted tumors in this study are EGFR negative^46^.

Our study indicates that tumor vessel normalization induced by axitinib is time and dose dependent. As axitinib has a restricted targeting spectrum and higher affinity for VEGF receptors than sunitinib, it may be argued that the induced vessel normalization effect seems to be VEGFR-related. The fact that we did not see enhanced oxygenation by the EGF targeted TKI erlotinib, further supports this suggestion. We have previously reported on the synergistic inhibition of tumor growth through the combination of sub-optimal doses anti-angiogenic TKIs with low-dose PDT^47^, showing that enhanced tumor growth inhibition may be achieved through the inhibition of PDT-induced angiogenic tissue responses, without the induction of vascular normalization. In this study we further investigated the intra-therapy time-dependent design of such a combination. PDT is a highly oxygen dependent therapy^8^, and its application during enhanced oxygenation period was found to improve the its anti-tumor effect. The delayed benefit in the PDT-related experiments may be due to the fact that PDT has been shown to induce angiogenesis that may counteract the effect of the angiogenesis inhibitor.

To date, the clinical efficacy in cancer patients of cytotoxic drugs combined with angiogenesis inhibitors is limited. As shown in a phase III trial, the treatment of newly diagnosed glioblastoma patients treated with bevacizumab prior temozolomide therapy did not improve the survival outcome^48^. Reduced perfusion and a reduced net influx rate of docetaxel in patients with NSCLC following bevacizumab therapy (15 mg/kg) was reported^24^. The feasibility of clinical applications of the normalization phenomenon (e.g. in a situation where the normalization window is induced for approximately 6 days^30^ and hyperfractionated radiotherapy is administered 5 times per week^49^) is widely discussed. It has been argued that in certain conditions there may even be a lack of real clinical benefit from vascular normalization, e.g. for the use of anti-VEGF antibody therapy in the case of cerebral tumors where normalization may reduce drug delivery by helping the re-establishment of brain microvasculature which is normally characterized by reduced permeability^50^. However, our studies confirm that vascular normalization and treatment benefit can be obtained with VEGFR-targeting small molecule-based inhibitors. Normalization can both enhance extravasation of small molecule chemotherapeutics to the intratumoral space, as well as enhance oxygen dependent treatment strategies. Our study also stresses the need for precise timing and scheduling of the combination treatment^51^. Further development of the encouraging results reported here in more advanced preclinical models will require extensive efforts to identify optimized dosages and timing schedules to maximally exploit the potential therapeutic benefits of such treatment strategies.

## Methods

### Cells lines and reagents

A2780 human ovarian carcinoma cells (ECACC, Salisbury, UK) were maintained in RPMI-1640 cell culture medium supplemented with GlutaMAX™ (Gibco, Carlsbad, USA), 10% heat-inactivated bovine calf serum (Sigma-Aldrich, St. Louis, USA) and 1% antibiotics (Sigma-Aldrich). Immortalized human vascular endothelial cells (ECRF24) were maintained in medium containing 50% DMEM and 50% RPMI 1640 supplemented with 1% of antibiotics (Life Technologies, Carlsbad, California, USA). ECRF24 were always cultured on 0.2% gelatin coated surfaces.

RAPTA-C ([Ru(η^6^-*p*-cymene)Cl_2_(pta)] (pta is 1,3,5-triaza-7-phosphaadamantane)) was synthesized and purified as described previously^52^. RAPTA-C was freshly prepared prior to use by dissolving power in DMSO at a concentration of 150 mg/ml. Axitinib was purchased from LC laboratories (Woburn, MA, USA) and dissolved in DMSO at a concentration of 20 mM at stored at −20°C for subsequent use. Doxorubicin HCl was acquired from Pharmachemie BV, Haarlem, The Netherlands, and was freshly dissolved in DMSO at 10 mg/ml. Drug dilutions were prepared in 0.9% NaCl solution for *in vivo* CAM assays or the appropriate cell medium for *in vitro* assays.

### In ovo pO_2_ measurements

Oxygen partial pressure (pO_2_) within tumors was recorded with an OxyLab pO_2_ meter (Oxford Optronix Ltd., Oxford, UK) coupled to a calibrated fiber optic probe (NP/O/E) placed in a 23 G surgical steel needle. The measurements were recorded 60 seconds after the insertion of the needle into the tumor core. pO_2_ measured in untreated tumors were compared to the measurements obtained in tumors treated with axitinib (4.5 or 36 μg/kg of axitinib), sunitinib (70 μg/kg) or erlotinib (50 μg/kg) injected on EDD 13 (t = 0) and followed by a second injection of the same drug dose on EDD 14 (t = 24 h).

### Fluorescence quantification

The CAM vasculature was visualised with a Nikon Eclipse E600FN epi-fluorescence microscope (Nikon AG, Tokyo, Japan) and a Plan Fluor objective 10 × 0.3 (working distance: 16 mm, Nikon AG). Intravenous injection of doxorubicin (1.5 mg/mL) induced the blood vessels fluorescence that enabled angiography. Contrast was improved with 100 μl injection of India ink into the extra-embryonic space (Pelikan, Witzikon, Switzerland). Fluorescence angiographies were taken every 30 seconds for 15 minutes. Fluorescence angiographies were analysed with ImageJ software (version 1.40 a; National Institutes of Health, Bethesda, MD, USA). The fluorescence quantification of doxorubicin distribution was performed by evaluating the average pixels value in the specified regions. To do that, three areas were established (within a blood vessel, within the tumour tissue and a peripheral background area) and the average pixel value in each of these regions was recorded at each of the desired time points. This data was then presented as the ratio of the average pixel value in the tumor tissue as compared to in the blood vessel, with both value corrected for the background by subtracting the average background fluorescence.

### Tumor growth inhibition

Fertilized chicken eggs were incubated in a hatching incubator (relative humidity 65%, 37°C), as previously described^53^. A2780 cells (10^6^) were prepared in serum-free RPMI-1640 as a spheroid in a 25 μl hanging drop and 3 h later were transplanted on the surface of the *in ovo* CAM on EDD 8^54^. Vascularized three-dimensional tumors were visible 3 days after tumor cell implantation, on EDD 11, when treatment was initiated. Tumors were treated daily by i.v. injection of freshly prepared compounds into a main blood vessel of the CAM, i.e. (i) axitinib, (ii) RAPTA-C (iii) doxorubicin and combinations of (i) and (ii), or (i) and (iii). Tumors were monitored daily and measured (volume = [larger diameter] × [perpendicular diameter]^2^ × 0.52). At the last experiment day embryos were sacrificed and tumors were resected and fixed in zinc-fixative for additional analysis. Low light fluence Visudyne®-PDT (1 J/cm^2^, 2.5 J/cm^2^, 5 J/cm^2^) was performed on EDD 11 (λ_ex_ = 420 nm, λ_em_ > 470 nm, irradiance of 28.5 ± 1.72 mW/cm^2^), as a single therapy, or at a dose of 2.5 J/cm^2^ in combination with axitinib (4.5 or 36 μg/kg) at t = 24 or 30 h after the initial axitinib injection on EDD11. When tumors were treated with PDT, the size of the diaphragm was adjusted so that the area of irradiation exactly corresponded to the visible tumor area.

### Tumor samples and immunohistochemistry

On the last day of the experiment, tumors were resected and fixed overnight in zinc fixative solution. CAM tumors were stained as previously described^26^. Briefly, 5 μm sections were blocked with 5% BSA in PBS followed by incubation with primary antibodies against CD31 (rat anti-CD31; 1:200, clone SZ31, Dianova, Hamburg, Germany). Secondary donkey anti-rat biotinylated antibodies (1:200; Jackson, Suffolk, UK) were then incubated, followed by Streptavidin-HRP (1:50; Dako, Glostrup, Denmark), and visualized by 3,3'-diaminobenzidine (DAB), resulting in a brown-colored precipitate at the antigen site.

### *In vitro* study on synergy between axitinib and doxorubicin or axitinib and RAPTA-C

Cell viability and migration assays were performed as previously described^25^. Cells were seeded in a 96-well culture plate at a density of 10 × 10^3^ cells/well 24 hours prior to drug administration. After applying compounds at their desired concentrations, cells were incubated with drugs for 72 h. Cell viability was assessed using the CellTiter-Glo luminescent cell viability assay (Promega, Madison, WI, USA).

### Statistical analysis

Values are given as mean values ± standard error of the mean (SEM) after independent experiments performed several times. Statistical analyses were done using the two-way ANOVA test for *in vivo* tumor growth studies or a two-tailed student t-test for other data. P-values < 0.05 were considered as being statistically significant.

## Author Contributions

A.W. performed *in vitro* and *in vivo* studies, statistical analyses, interpreted the data, and wrote the manuscript. D.B., R.H.B., E.S. and T.J.W. performed *in vivo* studies, image analysis, interpreted the data. P.J.D. participated in data interpretation and writing the manuscript. A.W.G. contributed to the study design, analyzed and interpreted the data and contributed in writing the manuscript. P.N.S. conceived and designed the study, performed and coordinated the *in vitro* and *in vivo* experiments, and wrote the manuscript. All authors read and approved the final manuscript.

## Supplementary Material

Supplementary Information Suplemantary info
