# Supplementary material for: Angiostatic treatment prior to chemo- or photodynamic therapy improves anti-tumor efficacy
Source: Sci Rep. 2015 Mar 11;5:8990. doi: 10.1038/srep08990 (PMC4355632; doi:10.1038/srep08990)
Supplement: Supplementary Information — Suplemantary info [file srep08990-s1.pdf]

Angiostatic treatment prior to chemo- or photodynamic therapy improves anti-tumor efficacy

Andrea Weiss[1,2], Débora Bonvin[1], Robert H. Berndsen[1,2], Edoardo Scherrer[1], Tse J. Wong[2], Paul J. Dyson[1], Arjan W. Griffioen[2], Patrycja Nowak-Sliwinska[1,2*]

[1]Institute of Chemical Sciences and Engineering, Swiss Federal Institute of Technology (EPFL), Lausanne, Switzerland; [2]Angiogenesis Laboratory, Department of Medical Oncology, VU University Medical Center Amsterdam, The Netherlands

Supplementary Figures

Figure S1.

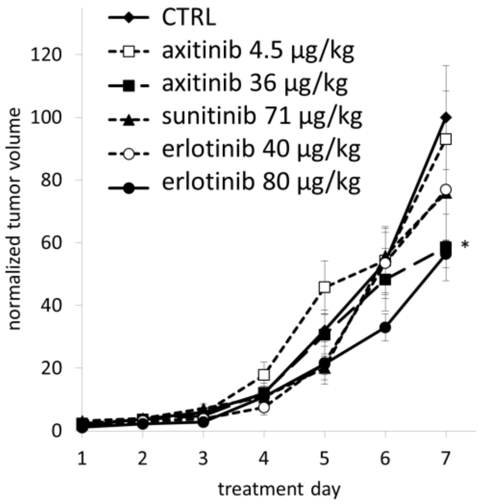

Figure S1. Efficacy of anti-angiogenic tyrosine kinase inhibitor therapy on tumor growth inhibition. Graph shows tumor volume as a function of treatment day and provided as a percentage of the control volume on the final experiment day for axitinib (4.5 µg/kg; n = 26; p = 0.74 and 36 µg/kg, n = 15; *p = 0.04), sunitinib (71 µg/kg; n = 9, p = 0.36), and erlotinib (40 µg/kg, n = 12; p = 0.44; 80 µg/kg, n = 12; p = 0.14). Values provided represent the mean tumor volume and error bars correspond to the standard error of the mean.

Figure S2.

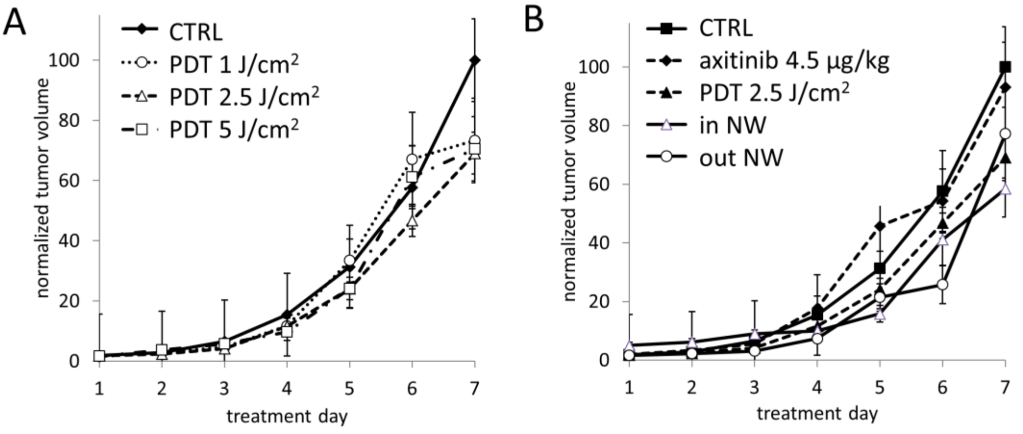

Figure S2. Efficacy of Visudyne®-PDT on tumor growth inhibition.

A. Tumor growth curves (shown as % control on the final experiment day) showing the effects of different doses of Visudyne®-PDT on tumor growth inhibition (0.2 mg per kg embryo weight; λ_{ex} = 420 ± 20 nm, drug light interval 1 min) at low light fluencies (1, 2.5 or 5 J/cm^2, irradiance 28.5 ± 1.7 mW/cm^2). **B.** Tumor growth curves showing the effects of Visudyne®-PDT (2.5 J/cm^2; n = 34), axitinib (4.5 µg/kg; n = 26) or the combination of both performed within ('in NW'; n = 7) or before ('out NW'; n = 15) the normalization window. Values provided represent the mean tumor volume and error bars correspond to the standard error of the mean.

Figure S3.

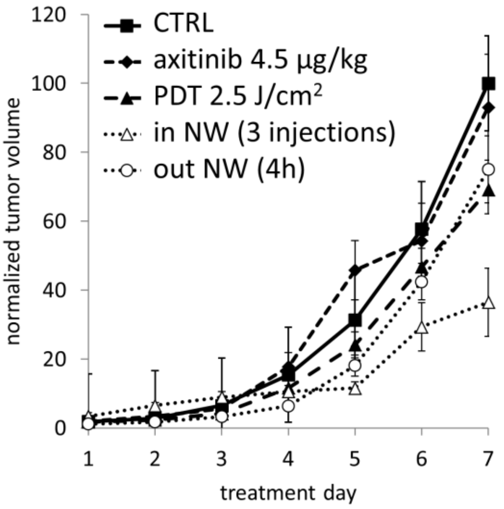

Figure S3. Efficacy of different treatment schedules involving Visudyne®-PDT on tumor growth. Tumor growth curves showing the effects of Visudyne®-PDT (2.5 J/cm^2; n = 34), axitinib (4.5 µg/kg; n = 26) or the combination of both at different treatment schedules: (i) Visudyne®-PDT performed 30 hours after the injection of axitinib (4.5 µg/kg) followed by a third axitinib injection 24 hours after PDT ('in NW (3 injections)'; n = 8), (ii) Visudyne®-PDT followed by two axitinib injections 4 hours and 24 hours after PDT ('out NW (4h)'; n = 10). Values provided represent the mean tumor volume error bars correspond to the standard error of the mean.

Table S1.

drug	dose (µM)	A2780 efficacy (%CTRL ± SEM)			ECRF24 efficacy (%CTRL ± SEM)		
axitinib	0.5	89 ± 4			90 ± 3		
	1	71 ± 4			80 ± 2		
RAPTA-C	100	99 ± 4			81 ± 4		
	200	93 ± 3			78 ± 6		
doxorubicin	0.01	85 ± 3			82 ± 3		
	0.05	66 ± 1			72 ± 3		
**combinations**			**CI**	**interaction**		**CI**	**interaction**
axitinib + RAPTA-C	1 + 100	70 ± 2	1	additive	66.2 ± 5	0.52	syngery
	1 + 200	71 ± 1	1.3	antagonistic	57.3 ± 8	0.38	syngery
axitinib + doxorubicin	1 + 0.01	65 ± 3	0.87	additive	96 ± 7	117	antagonistic
	1 + 0.05	75 ± 3	3.2	antagonistic	97 ± 2	1402	antagonistic

Table S1. Cell viability inhibition of axitinib, RAPTA-C, DOX or their combinations towards A2780 or ECRF24 cells. CI stands for combinatory index, where CI < 0.8 indicates synergy. Values are presented as means ± the standard errors of the mean (SEM).
